# Abnormal glucose tolerance in young male patients with nonalcoholic fatty liver disease

**DOI:** 10.1111/j.1478-3231.2008.01920.x

**Published:** 2009-04

**Authors:** Jung Won Yun, Yong Kyun Cho, Jung Ho Park, Hong Joo Kim, Dong Il Park, Chong Il Sohn, Woo Kyu Jeon, Byung Ik Kim

**Affiliations:** Department of Internal Medicine, Division of Gastroenterology, Kangbuk Samsung Hospital, Sungkyunkwan University School of MedicineSeoul, South Korea

**Keywords:** insulin resistance, nonalcoholic fatty liver disease, oral glucose tolerance test

## Abstract

**Objective:**

The association of nonalcoholic fatty liver disease (NAFLD) with insulin resistance and metabolic syndrome has been documented for obese men and middle-aged men. This study was designed to determine the relationship between NAFLD and the oral glucose tolerance test (OGTT) to predict preclinical diabetes in nondiabetic young male patients (<30 years old).

**Methods:**

A total of 75 male patients who had elevated liver enzymes and who were diagnosed with NAFLD were enrolled in this study. A standard 75 g OGTT was carried out on all patients. Impaired fasting glucose (IFG) and impaired glucose tolerance (IGT) were defined as a fasting plasma glucose (FPG) level ≥100 mg/dl but <126 mg/dl, and a 2-h post-load glucose on the OGTT of ≥140 mg/dl, but <200 mg/dl respectively.

**Results:**

According to the OGTT results, 24 (32%) patients were diagnosed as having IGT and 12 (16%) patients were diagnosed as having diabetes. Among the 48 patients with normal fasting glucose, 18 (37.6%) patients showed abnormal glucose tolerance (15 had IGT and three had diabetes). The NAFLD patients with abnormal glucose tolerance showed significant differences in age, weight, body mass index, waist–hip ratio, alanine aminotransferase, total bilirubin, total cholesterol, low-density lipoprotein cholesterol, triglyceride, insulin, FPG and homeostasis model for insulin resistance (HOMA-IR). Multiple regression analysis showed that age, FPG and HOMA-IR were independent predictors of abnormal glucose tolerance.

**Conclusions:**

Although the patients were young men, an OGTT should be recommended for NAFLD patients with elevated liver enzymes and IFG to predict the risk of type 2 diabetes.

Nonalcoholic fatty liver disease (NAFLD) covers a wide range of conditions from steatosis and nonalcoholic steatohepatitis (NASH) to cirrhosis and liver failure. The prevalence of NAFLD in western countries is at least 20% of the general population and it is up to 70% for obese patients or those with type 2 diabetes mellitus (DM) (1–4). Because of the increasingly westernized life style in Asian countries, NAFLD has become a major cause of liver disease and its prevalence is estimated to be up to 18% in Korea (5).

Nonalcoholic fatty liver disease is typically associated with central obesity, DM, hypertension, dyslipidaemia and metabolic syndrome (6–8). The pathogenic mechanism for NAFLD has been explained by the ‘two-hit’ theory, which is as follows:(i) accumulation of fat in hepatocytes and (ii) increased hepatic oxidative stress (9). Insulin resistance promotes fatty acid accumulation and oxidative stress (10). Ever since insulin resistance was first demonstrated in NAFLD patients (11), subsequent reports have shown that insulin resistance plays a key role in disease progression (12–14).

Patients with impaired glucose tolerance (IGT) and/or impaired fasting glucose (IFG) are now considered as being prediabetic, which indicates their relatively high risk for developing DM. IGT and IFG are associated with metabolic syndrome (15). It is important to identify patients with NAFLD in the stage of preclinical diabetes or glucose intolerance because they are at a high risk for developing DM and diabetes may be a risk factor for the development of progressive fibrosis (16).

The aim of this study was to evaluate the value of an oral glucose tolerance test (OGTT), in order to diagnose preclinical diabetes in nondiabetic young male NAFLD patients who are below 30 years of age.

## Materials and methods

### Patients

The study was prospectively conducted from April 2006 to April 2007 at our institution. The study was approved by the appropriate local ethics committees, and all patients provided written informed consent before enrollment in the study. All the enrolled patients who were referred to our outpatient clinic showed an elevated alanine aminotransferase (ALT) level and a fatty liver, as diagnosed by ultrasonography. Elevated ALT was defined as >40 IU/L. The diagnostic criteria for fatty change in the liver by ultrasonography were as follows (17): (i) a diffuse hyperechoic echotexture (bright liver), (ii) an increased liver echotexture compared with the kidney, (iii) vascular blurring and (iv) deep attenuation. Patients were excluded if they had a history of viral hepatitis, alcohol intake of 30 g or more per week, use of medication known to cause hepatic steatosis such as amiodarone, tamoxifen, methotrexate, tetracycline or corticosteroid and a previous history of DM.

### Clinical and laboratory analysis

The patients' age and family histories of DM were recorded. The systolic and diastolic blood pressures were measured in the sitting position after at least 10 min of rest. During the 30 min preceding the measurement, subjects were required to refrain from smoking or from consuming caffeine. The blood pressure was measured twice and the mean value was recorded. Anthropometric analysis included height, weight and body mass index (BMI), and the latter was calculated as weight (kg) divided by the square of the height (m^2^). The waist–hip ratio (WHR) was defined as the waist circumference (cm) divided by the hip circumference (cm). The waist and hip circumferences were measured in the horizontal planes at the levels of the umbilicus and the trochanter respectively. Laboratory analysis was performed via standard laboratory methods. The serum biochemical parameters included aspartate aminotransferase (AST), ALT, total bilirubin, γ-glutamyltransferase, alkaline aminotransferase, total cholesterol, low-density lipoprotein cholesterol (LDL-C), high-density lipoprotein cholesterol (HDL-C), triglyceride, fasting plasma glucose (FPG) and fasting insulin. Serum biochemistries were performed with a Bayer model 1650 automated bio-analyzer (Bayer Diagnostic, Basingstoke, UK). Serum insulin levels were measured with a solid-phase radioimmunoassay (Diagnostic Products Corporation, Los Angeles, CA, USA). The index of insulin resistance was calculated on the basis of fasting glucose and insulin by the homeostasis model for insulin resistance (HOMA-IR) (18).

### Definitions of the metabolic syndrome and the abnormal glucose tolerance test

Metabolic syndrome was defined by the new International Diabetes Federation definition (19): (i) Central obesity: waist circumference of 90 cm for Asian men and 80 cm or more for Asian women. (ii) Plus any two of four additional factors: (A) Raised triglyceride: >150 mg/dl, or specific treatment of this lipid abnormality. (B) Reduced HDL-C: <40 mg/dl in males and <50 mg/dl in females, or specific treatment of this lipid abnormality. (C) Raised blood pressure: systolic blood pressure ≥130 or diastolic blood pressure ≥85 mmHg, or treatment of previously diagnosed hypertension. (D) Raised FPG: 100 mg/dl or more, or previously diagnosed type 2 DM.

All the patients underwent a 75 g OGTT after an 8-h fast. Blood samples were obtained from all patients five times during the OGTT (before and 30, 60, 90 and 120 min after oral glucose loading). IFG and IGT were defined as an FPG level ≥100 mg/dl but <126 mg/dl, and a 2-h post-load glucose on the OGTT of ≥140 mg/dl, but <200 mg/dl respectively. DM was defined as a 2-h post-load glucose on the OGTT of >200 mg/dl (15). The definition of abnormal glucose tolerance included IGT or DM on the OGTT.

### Statistical analysis

The measured values are presented as median (range), and statistical analysis was performed using spss 11.0 (SPSS Inc., Chicago, IL, USA). Categorical variables were compared by the χ^2^-test or Fisher's exact test, whereas continuous variables were compared by the Mann–Whitney *U*-test. Significant variables from the univariate analysis were then subjected to multivariate stepwise logistic regression analysis to identify any independent significant factors associated with abnormal glucose tolerance among NAFLD patients. Time courses of the mean glucose concentrations during the OGTT were compared using repeated measures at different time points (0, 30, 60, 90 and 120 min). A *P*-value <0.05 was considered statistically significant.

## Results

**Fig. 1:**
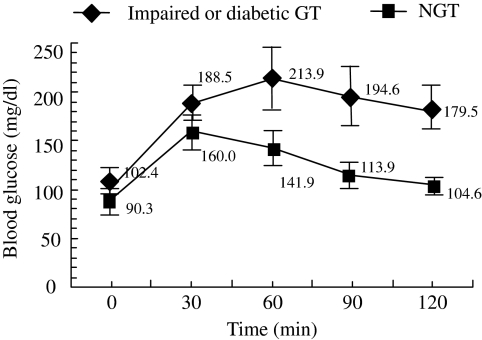
Time course of blood glucose levels during the oral glucose tolerance test (OGTT) between normal glucose tolerance (NGT) and impaired glucose tolerance/diabetic glucose tolerance. The mean glucose concentrations during the OGTT between the two groups were compared using repeated comparisons at different time points (0, 30, 60, 90 and 120 min). Data are presented as means with 95% confidence intervals. The differences of all time points (0, 30, 60, 90 and 120 min) were statistically significant respectively (*P*<0.05).

A total of 75 patients were enrolled in this study. The patients were all young (mean age: 23.5±3.7 years, range 19–30 years) males and slightly overweight (the mean BMI was 26.8±4.9 kg/m^2^ and the range was 18–41 kg/m^2^). Twenty-seven (36%) patients were diagnosed with IFG. According to the 75 g OGTT, 24 (32%) patients were diagnosed as having IGT and 12 (16%) patients were diagnosed as having diabetes. Among the 48 patients with normal fasting glucose, 18 (37.6%) patients showed abnormal glucose tolerance (15 had IGT and three had DM). The distribution of metabolic syndrome was 9/39 (23.1%) in normal OGTT, 6/24 (25%) in IGT and 9/12 (75%) in DM respectively (*P*<0.05). The clinical characteristics of the NAFLD patients with normal and abnormal OGTT are presented in Table 1. Comparing the demographical and anthropometrical variables, the patients with abnormal OGTT results were older and their weight, BMI and WHR were also significantly higher than patients with a normal OGTT. The biochemical analysis indicated that AST, ALT, total bilirubin, total cholesterol, LDL-C, triglyceride, fasting glucose, fasting insulin and HOMA-IR were significantly higher in patients with an abnormal OGTT. During the OGTT, significant differences in the plasma glucose levels were seen at 0, 30, 60, 90 and 120 min between the patients with a normal and an abnormal OGTT respectively (Fig. 1). Further analysis using multiple regression showed that age [odds ratio (OR): 2.172, 95% confidence interval (CI): 1.384–3.408], fasting glucose (OR: 1.197, 95% CI: 1.017–1.410) and HOMA-IR (OR: 3.374, 95% CI: 1.497–8.064) turned out to be independent predictors of abnormal glucose tolerance (Table 2).

**Table 2:** Predictors of abnormal glucose tolerance in nonalcoholic fatty liver disease patients according to oral glucose tolerance test by multivariate analysis

Variables	Odds ratio	95% CI	*P* value
Age (years)	2.172	1.384–3.408	0.001
Fasting glucose (mg/dl)	1.197	1.017–1.410	0.031
HOMA-IR	3.374	1.497–8.064	0.004

Multivariate logistic regression model adjusted for age, BMI, waist–hip ratio, AST, ALT, total bilirubin, total cholesterol, LDL-cholesterol, triglyceride, fasting glucose, fasting insulin and HOMA-IR.

ALT, alanine aminotransferase; AST, aspartate aminotransferase; BMI, body mass index; HOMA-IR, homeostasis model for insulin resistance; LDL, low-density lipoprotein.

**Table 1:** Characteristics of the study populations according to the oral glucose tolerance test results

Variable	NGT (*n*=39)	IGT or diabetic GT (*n*=36)	*P* value
Demographics			
Age (years)	21 (19–26)	26 (19–30)	<0.001
Family of DM (%)	18 (46.2)	18 (50)	NS
Systolic BP (mmHg)	114 (97–148)	116 (101–159)	NS
Diastolic BP (mmHg)	72 (65–90)	70 (62–88)	NS
Metabolic syndrome (%)	9 (23.1)	15 (41.7)	NS
Anthropometrics			
Weight (kg)	76 (61–96)	85 (67–129)	0.015
Height (cm)	175 (164–183)	175 (167–183)	NS
Body mass index (kg/m^2^)	24 (21–34)	28 (18–41)	0.010
Waist circumference (cm)	86 (76–109)	92 (75–125)	NS
Hip circumference (cm)	100 (95–114)	103 (80–125)	NS
Waist–hip ratio	0.88 (0.77–0.95)	0.92 (0.78–1.00)	0.024
Biochemical			
AST (IU/L)	44.5 (37–183)	60 (22–187)	0.024
ALT (IU/L)	84 (51–261)	117 (28–287)	0.001
T-bilirubin (mg/dl)	0.69 (0.41–1.64)	1.17 (0.71–2.23)	<0.001
ALP (IU/L)	74 (57–120)	76.5 (58–122)	NS
GGT (IU/L)	47 (16–149)	64.5 (20–134)	NS
Total cholesterol (mg/dl)	206 (150–245)	219 (160–322)	0.013
LDL cholesterol (mg/dl)	123 (87–173)	134.5 (109–201)	<0.001
HDL cholesterol (mg/dl)	43 (32–59)	38.5 (32–80)	NS
Triglyceride (mg/dl)	140 (76–404)	197.5 (108–564)	< 0.001
Oral glucose tolerance test (mg/dl)			
0-h plasma glucose	86 (77–114)	100 (88–119)	<0.001
120-min plasma glucose	110 (40–129)	155 (140–297)	<0.001
Fasting glucose (mg/dl)	86 (77–114)	100 (88–119)	<0.001
Fasting insulin (IU/ml)	8.2 (6.4–23.8)	13.85 (8.4–25.0)	<0.001
HOMA-IR	1.81 (1.24–6.40)	3.28 (2.09–5.43)	<0.001

The data were expressed as *n* (%) or median (range).

ALP, alkaline aminotransferase; ALT, alanine aminotransferase; AST, aspartate aminotransferase; BP, blood pressure; GGT, γ-glutamyl transferase; HDL, high-density lipoprotein; HOMA-IR, homeostasis model for insulin resistance; IGT, impaired glucose tolerance; LDL, low-density lipoprotein; NGT, normal glucose tolerance; NS, nonsignificant.

## Discussion

In the present study, we assessed, using the OGTT, for the early diagnosis of DM in male NAFLD patients with an elevated ALT level. Not many of our enrolled patients showed abnormal responses on the OGTT. Sixteen per cent of the patients were diagnosed with DM and 32% were diagnosed with IGT. The relationship between abnormal glucose tolerance and NAFLD has been established in several studies. A previous study of 73 patients (mean age: 45±8 years) with biopsy-proven NAFLD showed that 33 and 29% of the patients had DM and IGT respectively (20). Of 114 NAFLD patients (mean age: 53.6±10.5 years) who showed an elevated aminotransferase level, 50 patients showed abnormal glucose tolerance (IGT or DM) (21). In another study of 23 NAFLD patients, 3/13 patients (23.1%, mean age: 37.9±10.7 years) with abnormal liver enzymes were diagnosed with IGT and only one patient had DM, whereas none of the 10 patients with normal liver enzymes showed 2-h post-load glucose levels ≥140 mg/dl on the OGTT (22). As compared with the previous study, there were little differences in the prevalence of undiagnosed DM or IGT in the NAFLD patients. Yet the main difference in the present study was the relatively young age of the patients (mean age: 23.5±3.7 years, range: 19–30 years). Compared with the nondiabetic subjects, the patients with type 2 DM and NAFLD appear to have a higher risk of developing fibrosis and cirrhosis (10, 23, 24). Haukeland *et al.* (25) reported that abnormal glucose tolerance was higher among patients with NASH compared with patients with simple steatosis (72 vs. 49%) and higher in NAFLD patients with fibrosis compared with patients without fibrosis (76 vs. 49%). Therefore, in view of the high prevalence of abnormal glucose tolerance after an OGTT even in these young NAFLD patients, an early intervention for NAFLD patients with IGT to prevent progression of DM and hepatic fibrosis would be needed.

Type 2 DM develops from an imbalance between insulin sensitivity and insulin secretion. The earliest finding of type 2 DM is impairment in the body's response to insulin and this is termed insulin resistance (26). Insulin resistance is also a common part of the pathophysiology of NAFLD. In our study, an increased HOMA-IR, age and FPG were independent factors associated with abnormal glucose tolerance in NAFLD patients with elevated liver enzymes. Even the incidence of metabolic syndrome was higher in abnormal OGTT (41.7%) than normal OGTT (23.1%) but they did not show statistical significance (*P*=0.07). This result showed that NAFLD patients who were diagnosed with IGT or type 2 DM were more insulin resistant than the normal glucose tolerance group. IFG is determined by an increased hepatic glucose output and by an insulin secretory defect, but IGT is mostly associated with peripheral insulin resistance and it is more strongly associated with the features of metabolic syndrome than IFG (27). If the patients had combined IGT and IFG, then the risk of subsequent DM was higher than that for isolated IGT or isolated IFG. Because the prevalence of IGT is higher than the prevalence of IFG in NAFLD patients with elevated liver enzymes (28, 29), the OGTT can be used as a routine procedure for detecting DM in younger patients.

One of the limitations of our study was the small sample size, and we did not enrol a normal liver enzyme group. Therefore, further study with sufficient numbers of NAFLD patients should be performed to provide generalized prognostic information. Another limitation of our study was that the diagnosis of NAFLD was based on ultrasonography, and this may have overlooked those patients with a small amount of fat infiltration of the liver. Ultrasonography is the most common method for diagnosing NAFLD in clinical practice and it has a sensitivity and a specificity of 94 and 84%, respectively (30, 31), but it shows less sensitivity, particularly when the hepatic fat infiltration is <30% (32). However, a liver biopsy would have been impossible to perform routinely in the present study because of ethical problems.

In conclusion, the prevalence of abnormal glucose tolerance was 48% in the enrolled Korean young male patients who showed NAFLD and elevated liver enzymes. Although the study patients were young men, OGTT should be recommended for NAFLD patients with elevated liver enzymes and IFG to predict their risk of type 2 diabetes.
